# Multilevel analyses of on-demand medication data, with an application to the treatment of Female Sexual Interest/Arousal Disorder

**DOI:** 10.1371/journal.pone.0221063

**Published:** 2019-08-15

**Authors:** Rob Kessels, Jos Bloemers, Adriaan Tuiten, Peter G. M. van der Heijden

**Affiliations:** 1 Emotional Brain B.V., Almere, Flevoland, the Netherlands; 2 Utrecht Institute for Pharmaceutical Sciences and Rudolf Magnus Institute of Neuroscience, Utrecht University, Utrecht, Utrecht, the Netherlands; 3 Department of Methodology and Statistics, Utrecht University, Utrecht, Utrecht, the Netherlands; 4 Department of Social Sciences, University of Southampton, Hampshire, United Kingdom; Universidad de Tarapaca, CHILE

## Abstract

Data from clinical trials investigating on-demand medication often consist of an intentionally varying number of measurements per patient. These measurements are often observations of discrete events of when the medication was taken, including for example data on symptom severity. In addition to the varying number of observations between patients, the data have another important feature: they are characterized by a hierarchical structure in which the events are nested within patients. Traditionally, the observed events of patients are aggregated into means and subsequently analyzed using, for example, a repeated measures ANOVA. This procedure has drawbacks. One drawback is that these patient means have different standard errors, first, because the variance of the underlying events differs between patients and second, because the number of events per patient differs. In this paper, we argue that such data should be analyzed by applying a multilevel analysis using the individual observed events as separate nested observations. Such a multilevel approach handles this drawback and it also enables the examination of varying drug effects across patients by estimating random effects. We show how multilevel analyses can be applied to on-demand medication data from a clinical trial investigating the efficacy of a drug for women with low sexual desire. We also explore linear and quadratic time effects that can only be performed when the individual events are considered as separate observations and we discuss several important statistical topics relevant for multilevel modeling. Taken together, the use of a multilevel approach considering events as nested observations in these types of data is advocated as it is more valid and provides more information than other (traditional) methods.

## Introduction

Multilevel regression modeling, also known as mixed-effects modeling, hierarchical linear modeling, or random-effects modeling [1–4], is widely applied for the analysis of longitudinal data [5]. That is, multiple repeated measurements nested within individuals. In this paper, we advocate the use of multilevel modeling for the analysis of a specific kind of longitudinal multilevel data, namely unbalanced event data collected from clinical trials investigating the efficacy of on-demand medication.

On-demand medication, or *pro re nata* medication, is only taken when necessary. This is in contrast to medication that must be taken at set intervals. Trial data of on-demand medication intake are often observations of discrete events or episodes of when the medication was taken, including for example data on symptom severity/alleviation and information on day and time of the medication intake. Research into on-demand administration of medication have been conducted for erectile dysfunction [6–8], premature ejaculation in men [9, 10], female sexual dysfunction [11, 12], hemophilia [13], irritable bowel syndrome [14] and gastro-oesophageal reflux symptoms [15, 16], amongst others.

In this paper, we analyze the data of an on-demand drug which is intended to increase sexual satisfaction in women [12]. During this trial, patients were instructed to take the on-demand drug prior to an expected sexual event and subsequently evaluate the event using a questionnaire. From this questionnaire, we derive a score for sexual functioning by taking the sum of five Likert scale items. Furthermore, in the trial, there was a baseline establishment (BLE) period with no medication and an active treatment period (ATP), where patients were administered either the drug or a placebo at random. As the number of sexual events differed between patients, the number of observations differed between patients as well (intentionally unbalanced design). Data with this property can be easily coded into a hierarchical data structure of events (level 1) collected in patients (level 2), and subsequently analyzed by applying a multilevel analysis.

Traditionally, such data are aggregated before analyzing them. For the current data, this means that for each patient the sexual functioning scores of the individual events are averaged over the events observed in the BLE period and over the events observed in the ATP which results in two aggregated scores for each patient. The main interest is then focused on the interaction between the between-factor (drug/placebo) and the within-factor (BLE/ATP). Two statistical methods regularly applied to these aggregated data are repeated measures ANOVA or a multilevel analysis treating the aggregated scores as nested observations within patients. However, aggregating these data and subsequently analyzing them using either an ANOVA or a multilevel approach has important drawbacks which we will explain below.

The first drawback concerns missing data. Patients who report no sexual events during either the BLE period or the ATP have no aggregated sexual functioning score. When conducting the repeated measures ANOVA, this problem is generally handled by applying listwise deletion which involves deleting part of the data that was collected (complete case analysis). This missing data problem can be overcome by employing a multilevel analysis on the aggregated data such that with *N* patients having sexual events during both periods, there are N ×2 observations.

The second drawback of aggregating the data is concerned with the fact that each individual average sexual functioning score has a different standard error associated with it when the variance of the underlying event scores differs between patients and/or when the number of events per patient differs, which is usually the case. For example, suppose one patient reports three sexual events, each event evaluated with an identical sexual functioning score of 10. Another patient also reports three sexual events, but this patient evaluates these events with sexual functioning scores of 8, 10 and 12. When aggregating these scores, both patients end up having the same average of 10, while the standard errors differ. Also, in general, averages derived from more events will have a smaller standard error than averages derived from fewer events. When first averaging over all events and subsequently analyzing them using an ANOVA or multilevel approach described above, this additional information is not taken into account.

These drawbacks can be overcome if a multilevel analysis in which the individual events are separate observations nested within patients is applied. Besides solving these drawbacks, this approach has two additional advantages. The first advantage of this multilevel approach is that it enables the examination of potentially varying drug effects across each individual patient by estimating a random drug effect. In clinical trials, the average drug effect is of main interest, but examining varying drug effects across individual patients can offer a valuable contribution to the evaluation of clinical trial results. Especially given the rise in personalized medicine, where the drug is tailored to the individual patient, it becomes more important to understand how a treatment effect varies across patients [17]. As such, the ability to include random drug effects in the multilevel model makes the model more flexible in evaluating drug trials than the ANOVA model, where random drug effects cannot be estimated. Furthermore, we note that estimating random drug effects in a multilevel model using the two aggregated scores is not possible because with only two observations per patient, this random effect parameter becomes unidentifiable [18].

The second additional advantage of using the individual events in a multilevel model is that other additional information available on the event-level can be included. For example, when considering the example described above, the patient having sexual function scores of 8, 10, 12 shows a growth over time. A repeated measures ANOVA or a multilevel analysis on the two aggregated scores does not enable the inclusion of this information in the analysis which means that this growth is ignored.

Unfortunately, a multilevel analysis in which the individual events are used as separate observations nested within patients is rarely applied to the analysis of on-demand medication data. A reason for this could be the relative complexity of the aforementioned method compared to the more conventional methods such as the ANOVA. Also, companies developing drugs may be more hesitant to use more modern techniques because regulatory agencies such as the U.S. Food and Drug Administration (FDA) are apparently more cautious of novel or alternative approaches [19].

There are two approaches in which multilevel models can be used to analyze repeated measurement data [20]. One approach is concerned with modeling the correlation structure of the repeated measurements within patients, thereby focusing on the within-patient variance-covariance structure. Instead of assuming a constant correlation across all measurements of the same patient, this approach allows for modeling an alternative correlation structure. For example, when specifying an autoregressive structure it is assumed that measurements close together in time have a larger correlation than measurements that are further apart, which often is more realistic than assuming all correlations are equal. This approach can be used for analyzing longitudinal clinical trial data where an outcome is repeatedly measured over time at planned intervals [21–23]. The other approach is concerned with estimating random effects across patients, thereby focusing on the variance-covariance structure of the random components. Which method to select depends on the goal of the research question and data characteristics. When the repeated measurements are unbalanced, as is the case with the on-demand medication data that are analyzed in this paper, modeling an alternative correlation structure is not feasible [18]. Therefore, in this paper we present a multilevel modeling approach including random effects as this is considered the best multilevel approach for analyzing unbalanced repeated measurement data [18]. As outlined before, the inclusion of random effects allows for exploring potentially varying treatment effects across patients, which may be valuable for all kinds of longitudinal data. We therefore believe the paper will be relevant for all researchers interested in analyzing data of longitudinal clinical trials.

The goal of this paper is to show how on-demand medication data can be analyzed by applying multilevel modeling techniques that use individual events as separate nested observations. First, we compare a mixed between-within-subjects (BWS) ANOVA, a multilevel model applied to the aggregated data and a multilevel model where the individual events are considered. Subsequently, we explore a different model in which we study the change over time using the contribution of a variable collected on the event level.

## Methods

### Data

In this paper, we use data from a double blind, placebo-controlled randomized clinical trial investigating the efficacy of on-demand use of the combined administration of testosterone and sildenafil (T+S), compared to placebo, in American women diagnosed with Hypoactive Sexual Desire Disorder (HSDD; which currently is part of the diagnosis Female Sexual Interest/Arousal Disorder [FSIAD]) caused by low sensitivity of the brain to sexual cues (Trial registration: www.ClinicalTrials.gov: ID: NCT01432665) [12]. In this study, patients were instructed to take the medication prior to an anticipated sexual event and they were instructed to report each event using an event diary during three study periods: a four-week BLE period, a single-blind eight-week placebo run-in (PRI) period, and a double-blind eight-week ATP. During the BLE period, no medication was administered and during the PRI period all patients received placebo. During the ATP, patients were randomized to one of seven treatment conditions where they could either receive placebo, sildenafil, testosterone or one of four different T+S dosages. As this paper presents a methodological exercise that is intended to show that event data must be analyzed using the events as observations, we will only use data of patients randomized to placebo (N_P_ = 27) or the T+S highest dose combination (N_T+S_ = 26), resulting in a total sample size of N = 53.

Data were collected using the validated sexual event diary (SED) [24], which is a web-based diary that patients had to fill out within 24 hours following a sexual event. Patients had to indicate if study medication was used, if the event was satisfactory (1 = *yes*, 0 = *no*), and the amount of sexual desire, pleasure, bodily arousal, subjective arousal, and inhibition that they had experienced during the event on a five-point Likert scale (1 = *not at all*, 5 = *totally*). The outcome variable used in the analyses presented in this paper was the total sexual function score for each event separately calculated by taking the sum of the five Likert-scale items. This approach is justified by the results of an exploratory factor analysis performed on these five Likert-scale items using 1495 events reported by these 53 women during the BLE, PRI, and ATP, including events without medication intake. Because of the ordinal scale of the items, the exploratory factor analysis was conducted on the polychoric item correlation matrix. A parallel analysis with a weighted least squares estimation method was performed to determine the number of factors. The results of this parallel analysis showed one factor should be retained. The eigenvalue of the first factor was 4.05 and explained 76% of the variance and all other eigenvalues were far below one. The five items had very strong factor loadings (≥ .78) on this one factor confirming that a one-factor structure was an adequate solution. This factor could be interpreted as a factor measuring the total sexual functioning of a single event which was then derived by taking the sum of the five SED Likert-scale items. Furthermore, the Cronbach’s Alpha coefficient, that provides a lower-bound for the reliability of the five SED items, was equal to 0.94. This indicates the reliability of the items is very good and more than sufficient to sum the items to a scale.

### Data preparations

For conducting the different analyses in this paper, the data were coded in the appropriate formats and only the events reported during the BLE period and ATP were included. In the design, the PRI period was included to stabilize any potential placebo effects and interest was focused on the change in sexual functioning from BLE to ATP. Furthermore, only the events following medication intake during the ATP were included. As a result, 627 events were included, of which 229 events were reported during the BLE and 398 events during the ATP.

To derive the aggregated sexual function scores for the BLE and ATP, the amount of sexual functioning belonging to each event was averaged across the total number of events observed during the four weeks of the BLE period and during the eight weeks of the ATP. A “wide data” representation for two patients is listed in Table 1A, where each row represents a patient having a sexual function score for the BLE period and for the ATP. The total number of satisfying sexual events and unsatisfying sexual events during both periods is also included to illustrate that the sexual function scores are averages over satisfying and unsatisfying events. The treatment group is included as a binary indicator (0 = *placebo*, 1 = *T+S*).

**Table 1:** Wide (A) and Long (B,C) data formats for presenting the data of two patients.

**A**							
ID	Treatment group	SSEs BLE	SSEs ATP	USEs BLE	USEs ATP	SF BLE	SF ATP
8	1	1	12	3	0	8.50	21.08
11	0	2	2	3	4	9.40	8.83
**B**							
ID	Treatment group	Study period	SSEs	USEs	SF		
8	1	0	1	3	8.50		
8	1	1	12	0	21.08		
11	0	0	2	3	9.40		
11	0	1	2	4	8.83		
**C**							
ID	Treatment group	Study period	Event count	Satisfied	SF		
8	1	0	0	0	10		
8	1	0	1	0	6		
8	1	0	2	0	7		
8	1	0	3	1	11		
8	1	1	0	1	19		
8	1	1	1	1	17		
8	1	1	2	1	25		
8	1	1	3	1	25		
8	1	1	4	1	25		
8	1	1	5	1	25		
8	1	1	6	1	19		
8	1	1	7	1	20		
8	1	1	8	1	17		
8	1	1	9	1	23		
8	1	1	10	1	23		
8	1	1	11	1	15		
11	0	0	0	0	8		
11	0	0	1	1	13		
11	0	0	2	0	6		
11	0	0	3	1	13		
11	0	0	4	0	7		
11	0	1	0	0	5		
11	0	1	1	0	6		
11	0	1	2	0	5		
11	0	1	3	1	15		
11	0	1	4	1	12		
11	0	1	5	0	10		

Abbreviations: SSE = Satisfying Sexual Event, USE = Unsatisfying Sexual Event, BLE = Baseline Establishment, ATP = Active Treatment Period, SF = Sexual Functioning.

The data from Table 1A were transformed into a “long data” format. This procedure involves two steps. In the first step, the variables “Sexual Functioning BLE” and “Sexual Functioning ATP” were restructured into cases. This means that each row represents a BLE or an ATP average score. The data in this format is listed in Table 1B. An additional variable study period is now included to indicate if the score belongs to the BLE period (0) or ATP (1). This format is used for a multilevel analysis with the two aggregated averages nested within patients. For conducting the multilevel analysis using the individual events, a second step was required. This step involves splitting out the events for each period. The results of this process are listed in Table 1C. Here, each row represents a single event with the corresponding satisfaction score (0 = *Unsatisfied*, 1 = *Satisfied*) and sexual function score. In contrast with the format presented in Table 1B, this representation allows for the inclusion of a time variable. Here we use the event count by simply counting the number of events starting at zero. Event count is used to investigate if women with sexual problems show a learning effect over time (i.e. trend analysis) in their sexual functioning scores once they receive a drug that is expected to improve their sexual satisfaction. This theorem is based on the fact that sexual behavior is largely learnt. Early sexual interactions have a strong influence on sexual behavior later in life [25], but also later in life each sexual interaction can influence subsequent interactions, especially if these are particularly positive or negative [26]. To determine whether the learning effect is different between a period when there is no medication intake (BLE) and a period when patients receive medication (ATP), the variable event count starts at zero when the BLE and the ATP begin. Event count, study period, and satisfaction (binary variable indicating whether the event was satisfactory) are time-varying covariates varying at the event-level and therefore located at the event-level. On the other hand, because each patient receives either placebo or T+S, the variable treatment group varies only between patients and is therefore located at the patient-level.

### Models

Before we introduce the regression equations, we shall make a distinction between fixed and random regression coefficients. Fixed coefficients are constant across individuals while random coefficients vary across individuals [27]. In the full multilevel model, the intercept and slope have a fixed component and a random component. The random component indicates the degree to which a fixed effect varies at the second level (i.e. over patients). The amount of variation is represented by the size of the random component.

#### Model for analyzing aggregated data

Let *Y*_*ti*_ be the sexual function outcome for person *i* at the measurement instance *t*, with *i* = 1,…,*N* and *t* = 1,2 for the BLE score and ATP score, respectively. Furthermore, let *X*_*ti*_ be the {0, 1} dummy coded variable study period at measurement instance *t* for patient *i* and let *Z*_*i*_ be the {0, 1} dummy coded variable treatment group for patient *i*. We start by considering a multilevel model with only a random term for the intercept. This model is defined as shown below.

$$\begin{array}{rr} \text{Level}\;1:Y_{ti} & =\beta_{0i}+\beta_{1i}X_{ti}+\varepsilon_{ti}\\ \text{Level}\;2:\beta_{0i} & =\gamma_{00}+\gamma_{01}Z_{i}+u_{0i}\\ \beta_{1i} & =\gamma_{10}+\gamma_{11}Z_{i}\\ \text{Single}\;\text{equation}:Y_{ti} & =\gamma_{00}+\gamma_{10}X_{ti}+\gamma_{01}Z_{i}+\gamma_{11}Z_{i}X_{ti}+u_{0i}+\varepsilon_{ti}, \end{array}$$ (1)

where the patient-specific random intercept *β*_0*i*_ is modeled as a function of the overall intercept *γ*_00_, that represents the average sexual function score at baseline for patients receiving placebo, plus the difference from this mean for patients receiving T+S, *γ*_01_, and the random intercept term *u*_0*i*_ for patient *i*. The random intercept *u*_0*i*_ has a mean equal to zero and variance $\sigma_{u0}^{2}$. This variance reflects the degree to which the intercepts vary over the patients. The residual error term, *ε*_*ti*_, has a mean of zero and variance $\sigma_{\varepsilon }^{2}$, which is the variance of the first-level residuals.

The slope for study period, *β*_1*i*_, is modeled as a function of the fixed slope for the placebo patients, *γ*_10_, plus a deviation from this slope for the T+S patients, denoted by *γ*_11_. This parameter defines a cross-level interaction effect between study period and treatment group.

Model (1) applied to the aggregated data using complete cases only is analogous to a BWS ANOVA, as a model with only a random intercept assumes compound symmetry [2]. Compound symmetry requires that all population variances of the repeated measures are equal and that all population covariances of the repeated measures are equal, which is the same restriction present in repeated measures ANOVA [28].

The difference between the BWS ANOVA and a multilevel model applied to the aggregated data presented in Table 1B, is that the multilevel approach can include complete and incomplete cases.

#### Model for analyzing individual events

As described in the Introduction section, we advocate a multilevel analysis of the individual events as the preferred approach, because it considers the different underlying standard errors associated with the averages over the events, it enables the examination of varying drug effects across patients and it allows for the inclusion of additional variables located at the event-level.

The multilevel model for the analysis of the individual events can be described by the following set of equations.

$$\begin{array}{rr} \text{Level}1:Y_{ei} & =\beta_{0i}+\beta_{1i}X_{ei}+\varepsilon_{ei}\\ \text{Level}2:\beta_{0i} & =\gamma_{00}+\gamma_{01}Z_{i}+u_{0i}\\ \beta_{1i} & =\gamma_{10}+\gamma_{11}Z_{i}\;u_{1i}\\ \text{Single}\;\text{equation}:Y_{ei} & =\gamma_{00}+\gamma_{10}X_{ei}+\gamma_{01}Z_{i}+\gamma_{11}Z_{i}X_{ei}+u_{0i}+\varepsilon_{ei}. \end{array}$$ (2)

The outcome variable is now written as *Y*_*ei*_, which is the evaluation of event *e* by patient *i*. Similarly, study period is now denoted as *X*_*ei*_, as it varies over the events and the residual error term is now written as *ε*_*ei*_. In Model (2), all fixed *γ* parameters have the same interpretation as in Model (1). The level 2 equation of Model (2) includes a random slope for study period *β*_1*i*_, in addition to a random intercept *β*_0*i*_. This patient-specific random slope is modeled as a function of the fixed study period slope for the placebo patients, *γ*_10_, plus a deviation from this slope for patients receiving T+S, denoted by *γ*_11_, and the random slope term *u*_1*i*_ for patient *i*, that has a mean of zero and variance $\sigma_{u1}^{2}$. In this model, the slope variance indicates the degree to which the fixed effect of study period varies across patients, after the differences due to the treatment group between patients are considered. Thus, this model allows for individual differences in the effect of the placebo and the active drug.

#### Models for trend analyses: The learning effect

In many studies that collect longitudinal data, the interest is focused on the trend, development, growth, or change over time of certain behaviors. For researchers that aim to model such trends, growth curve analyses are an effective analytical solution that can be performed using multilevel modeling [5]. In this paper, we are interested in the learning effect of sexual behavior. To model this learning effect, we extended Model (2) with a linear term and with a linear plus a quadratic term for the event count variable presented in Table 1C. The ability to include these linear and quadratic terms is another reason to apply the multilevel approach on the individual events. As discussed before, to determine whether the learning effect is different between the BLE period and ATP, the variable event count restarts at zero when the ATP begins and interaction effects between study period and the linear and/or quadratic term for event count can be included to model these differences.

For this analysis, event count is denoted as *T*_*ei*_, starting at *t*_1*i*_ = 0 for the first event of the BLE and for the first event of the ATP, for patient *i*. When considering Model (2), including a fixed linear effect results in *Y*_*ei*_ = *β*_0*i*_ + *β*_1*i*_*X*_*ei*_ + *β*_2_*T*_*ei*_ + *ε*_*ei*_ at the first level with *β*_2_ = *γ*_20_ as additional second level equation. Including a fixed quadratic effect of the event count results in $Y_{ei}=\beta_{0i}+\beta_{1i}X_{ei}+\beta_{2}T_{ei}+\beta_{3}T_{ei}^{2}+\varepsilon_{ei}$ at the first level with *β*_3_ = *γ*_30_ as additional second level equation.

### Distinguishing between within-patient and between-patient effects in first-level variables

In multilevel models, a first-level variable consists of within-patient variation and between-patient variation. This means that the fixed effect of a first-level variable is a mix between a within-patient effect and a between-patient effect. This can be problematic, for example, when someone wants to evaluate the change within a patient. Then it is undesirable that this within-effect is confounded with the between-effect. One way to distinguish between the within-patient and between-patient effect is to add the patient-means as second-level predictor to the model [29]. By using this approach, the model provides the within-patient effect and the difference between the within-patient and between-patient effect. Thus a test whether the regression coefficient of the second-level patient means predictor is equal to zero is equivalent to testing whether the within and between effects are equal.

As an additional analysis, we explored any differences between the within-patient and between-patient effects of the first-level variables study period and event count by adding the patient means as predictors of the random intercept at the second level. Consider the model including the cross-level interaction between study period and treatment group, the random slope of study period and the fixed linear and quadratic terms for the event count. Let $\bar{X_{i}}$ be the mean of the dichotomous predictor study period for patient *i*, which represents the proportion of ATP events. Because the random intercept is interpreted as the average value of the outcome variable when all predictors are equal to zero, $\bar{X_{i}}$ was grand-mean centered by subtracting the overall average of the patient means from all values such that the score zero represents a patient with an average proportion of ATP events.

The mixing-up of between-patient and within-patient effects is also present in the cross-level interaction between study period and treatment group. This means the interaction is a combination of an interaction between the within-patient effect of study period and treatment group and an interaction between the between-patient effect of study period and treatment group. To test whether these effects are different, we included an interaction effect between the patient means of study period and treatment group at the second level and the regression coefficient of this interaction effect represents that difference.

Furthermore, $\bar{T_{i}}$ and ${\bar{T_{i}}}^{2}$ represent the grand-mean centered event count average and the squared grand-mean centered event count average for patient *i*. Patient means of event count can be interpreted as the weighted average number of events between the BLE and ATP. Including these additional variables yields the following set of equations.

$$\begin{array}{rr} \text{Level}1\;\text{:}\;Y_{ei} & =\;\beta_{0i}\;+\;\beta_{1i}X_{ei}\;+\;\beta_{2}T_{ei}\;+\;\beta_{3}T_{ei}^{2}\;+\;\varepsilon_{ei}\\ \text{Level}\;2\;:\;\beta_{0i} & =\;\gamma_{00\;}+\;\gamma_{01}Z_{i\;}+\;\gamma_{02}\bar{X_{i}}\;+\;\gamma_{03}\bar{X_{i}}Z_{i}+\;\gamma_{04}\bar{T_{i}}\;+\;\gamma_{05}\bar{T_{i}}{\;}^{2}+\;u_{0i}\\ \beta_{\text{1i}} & =\;\gamma_{10\;}+\;\gamma_{11}Z_{i}\;+\;u_{1i}\\ \beta_{2} & =\;\gamma_{20\;}\\ \beta_{3} & =\;\gamma_{30\;}\\ \text{Single}\;\text{equation}\;:\;Y_{ei} & =\;\gamma_{00\;}+\;\gamma_{10}X_{e}{}_{i\;}+\;\gamma_{20}T_{ei}\;+\;\gamma_{30}T_{ei}^{2}\;+\;\gamma_{01}Z_{i}\;+\;\gamma_{02}\bar{X_{i}}\;\\ + & \gamma_{03}\bar{X_{i}}Z_{i}\;+\;\gamma_{04}\bar{T_{i}}\;+\;\gamma_{05}\bar{T_{i}}{\;}^{2}\;+\;\gamma_{11}Z_{i}X_{ei}\;+\;u_{1i}X_{ei}\;+\;u_{0i}\;+\;\varepsilon_{ei.} \end{array}$$ (3)

where *γ*_10_ represents the within-patient effect of study period and *γ*_02_ is the difference between the within-patient and between-patient effect of study period. The same applies to *γ*_20_ and *γ*_04_ for the linear term of the event count and *γ*_30_ and *γ*_05_ for the quadratic term of the event count. Regarding the cross-level interaction: *γ*_11_ represents the cross-level interaction between the within-patient effect of study period and treatment group and *γ*_03_ is the difference between the cross-level interaction between the within-patient effect and treatment group and the interaction between the between-patient effect and treatment group.

### Statistical analysis

Analyses in this paper were performed using the open-source statistical software R, version 3.5.3 [30]. The multilevel analyses were conducted using the lme4 package, version 1.1-21 [31], together with the lmerTest package, version 3.1-0 [32]. All R-code used to generate the results can be found in S1 Appendix. For estimating the parameters of the models, we employ Full Maximum Likelihood (FML) estimation rather than Restricted Maximum Likelihood (REML) estimation. It should be noted that REML estimation provides less biased estimates for the variance components compared to FML estimation with smaller sample sizes [33]. The sample size in our analyses (N = 53) actually requires REML estimation, but for some multilevel models, REML estimation did not converge. For other models, the difference between the results for FML estimation and REML estimation were small. Therefore, the parameter values presented in this paper were estimated using FML estimation. An advantage of FML estimation over REML estimation is that FML estimation allows for the comparison of different models that differ in the fixed and random part using the deviance difference test (explained below).

In multilevel analysis, significance testing and model comparison is performed for the fixed regression coefficients and variance components in order to find the best fitting model. Maximum likelihood estimation produces standard errors that can be used to test the significance of the fixed regression coefficients by dividing the estimate by its standard error. The resulting test statistic is known as the Wald test and is usually evaluated against the normal distribution. It has been argued to evaluate the fixed regression coefficients against a *t*-distribution, as this is more conservative than evaluating against the normal distribution, especially with small sample sizes [1]. When using the *t*-distribution, an approximation of the degrees-of-freedom is required. In our analyses, we apply the Satterthwaite approximation for calculation of the degrees-of-freedom [34]. The Satterthwaite approximation is based on the two-group *t*-test formula given unequal residual variances and unequal sample sizes across the groups where the standard error in the formula weights the residual variance in each group by the sample size. In the multilevel model, a similar process is used, in which the degrees-of-freedom are estimated taking into account the variance components at each level of the hierarchy (i.e. events and patients), together with the first level and second level sample sizes. It has been shown the Satterthwaite approximation for calculation of the degrees-of-freedom performs well under small sample size conditions and unbalanced data in multilevel models [35]. By activating the lmerTest package before performing the analyses using lme4 functions, the output of the lme4 functions includes *p*-values for the *t*-tests of the fixed regression parameters based on the Satterthwaite approximation for the calculation of the degrees-of-freedom. If applicable, fixed effects will be evaluated for significance using a two-sided test with an alpha level of 0.05.

The required sample size for a multilevel analysis is dependent upon many factors, such as the number of levels, design of the study, scale of the dependent variable, whether interest is primarily focused on first-level fixed effects, second-level fixed effects, cross-level interactions or random effects, and ultimately, which question the researcher is asking. An excellent overview and discussion of sample size considerations for multilevel analyses is given by Hox et al. [2].

Testing the significance of variance components is often only of interest when exploratory research is performed to build the best fitting multilevel model. For testing the significance of the random slope variance, the Wald test is not appropriate as variance parameters are often not normally distributed [36]. As an alternative, a chi-square test on the residuals has been proposed to test these variance components [1]. In pharmaceutical research, however, the model of interest has already been determined by the design of the study. Exploratory analyses on the significance of the random parameters are therefore not of interest. In this paper, we are interested in a model including the cross-level interaction between study period and treatment group, a random intercept and a random slope for study period and the main goal is to test the cross-level interaction of the complete model using the *t*-test approach described above.

The complete model fit can be examined using the deviance. The deviance is derived as -2 x the log-likelihood, which is the natural logarithm of the likelihood function after reaching convergence. The deviance is used for model comparison and two models can be compared statistically using their deviances assuming that these models are nested. A model is nested under a more general model if it can be obtained from the general model by removing some parameters. The difference in deviances has a chi-square distribution with degrees-of-freedom equal to the difference in the number of estimated parameters. A non-significant chi-square test indicates the model with less parameters does not fit worse and should be preferred. This test is often referred to as a deviance difference test or a likelihood-ratio-test. As described before, when FML estimation is employed, the deviance difference test can be used for comparing two nested models that differ in their fixed and/or random part. In this paper, we will fit some additional models by adding first-level and second-level variables and we will use the deviance difference test to compare these nested models.

For comparing two non-nested models, Akaike’s Information Criterion (AIC) [37] can be used as a fit statistic. The AIC in multilevel models can be derived using the deviance as *deviance* + 2*q* where *q* is the number of estimated parameters in the model. Thus the AIC includes a penalty for the number of parameters which means the larger the number of parameters, the larger the value of the AIC. Lower AIC values are generally considered better model fits. The AIC is also a convenient method for comparing several different models, whether or not the models are nested and we will use the AIC as well to compare multiple models.

## Results

In the first part of the Results section, we discuss and compare the results of the BWS ANOVA and the multilevel model applied to the aggregated data and the multilevel model with the individual events as first level variables. In the second part, we present the results of the trend analyses. We finish the Results section by discussing the results of Model (3).

### Comparison of BWS ANOVA and multilevel regression models

#### Analysis on aggregated data

As described in the Methods section, when applying Model (1) to the aggregated data using complete cases only, the model is analogous to a BWS ANOVA. We therefore apply Model (1) to the data listed in Table 1B. There were six patients with a missing aggregated score on the BLE or ATP resulting in N = 47 complete cases. Traditionally, a BWS ANOVA is analyzed using the data format in Table 1A, where *F*-tests of the within and between-subject effects are generated. For a detailed discussion regarding the calculations of these *F*-tests, see Maxwell and Delaney [28].

The first column in Table 2 lists the results of the BWS ANOVA. The random intercept variance is 13.47, which indicates there is substantial variation in the baseline sexual function scores. Using the square root of this variance estimate, we find that approximately 67% of the individual patient intercepts are predicted to lie between one standard deviation below and one standard deviation above the mean intercept, also known as the predictive interval of the individual intercepts [2 p. 16]. The square root of 13.47 = 3.67, indicating that approximately 67% of the placebo patients have an estimated intercept between 13.24–3.67 = 9.57 and 13.24 + 3.67 = 16.91. For T+S patients the estimated intercept is 13.24–1.13 = 12.11, indicating that approximately 67% of the T+S patients have an estimated intercept between 12.11–3.67 = 8.44 and 12.11 + 3.67 = 15.78. The fixed effect parameters can be interpreted as follows: placebo patients have a baseline sexual function score of 13.24 and show an increase of 3.12 during the ATP. T+S patients have a baseline sexual function score of 12.11 and show an increase of 3.12 + 2.54 during the ATP. The only significant effect is the increase of 3.12 (*t* = 3.277, *p* = 0.002). The additional increase in sexual functioning of the T+S group compared to placebo during the ATP (cross-level interaction effect) is 2.54 (*t* = 1.860, *p* = 0.069). This result reveals no significant effect.

**Table 2:** Estimated parameters for BWS ANOVA and Multilevel models.

Model	BWS ANOVA		Multilevel models					
			On average scores		On individual events		On individual events with cov	
Fixed part (multilevel notation)	Coef (SE)	*p*-value	Coef (SE)	*p*-value	Coef (SE)	*p*-value	Coef (SE)	*p*-value
Intercept (*γ*_00_)	13.24 (1.01)		13.18 (0.99)		13.18 (0.87)		13.64 (1.09)	
Study period (*γ*_10_)	3.12 (0.95)	0.002	3.01 (0.94)	0.002	2.97 (0.94)	0.003	2.98 (0.95)	0.003
Treatment group (*γ*_01_)	-1.13 (1.44)	0.434	-1.73 (1.40)	0.219	-1.76 (1.25)	0.166	-1.64 (1.26)	0.197
Study period×Treatment group (*γ*_11_)	2.54 (1.36)	0.069	2.71 (1.35)	0.050	2.88 (1.36)	0.039	2.84 (1.36)	0.042
Age (*γ*_02_)							0.06 (0.09)	0.494
BMI (*γ*_03_)							0.03 (0.08)	0.706
Menopausal status (*γ*_04_)							-1.48 (2.06)	0.477
**Random part**								
$\sigma_{\varepsilon }^{2}$	10.91		10.96		9.39		9.39	
$\sigma_{u0}^{2}$	13.47		13.97		17.17		17.06	
$\sigma_{u1}^{2}$					18.01		18.08	
*r*(*u*_0_,*u*_1_)					-0.25		-0.27	
**Deviance**	549.9		587.7		3411.5		3410.6	
**AIC**	561.9		599.7		3427.5		3432.6	

Abbreviations: Coef = Coefficient, SE = Standard error, cov = covariates, AIC = Akaike Information Criterion

For performing a multilevel model on the aggregated scores, Model (1) was also applied to data listed in Table 1B including the patients with a missing value on one of the two time points (see the second column of Table 2 for the results of this analysis). The fixed effects do differ slightly from the fixed effects of the BWS ANOVA. These differences can be explained by the fact this multilevel model includes six patients having incomplete data. This multilevel model cannot estimate a random slope, because then the number of random effects (two, namely for intercept and slope) is equal to the number of observations per patient. As a result, the slope variance is confounded with the residual variance leading to a variance-covariance matrix that is unidentified [18]. When considering the model using the individual events, there are no such identification problems.

#### Analysis on individual events

For this analysis, we apply Model (2) to the data listed in Table 1C. The results of this analysis are listed in the third column of Table 2. The fixed parameters do not differ substantially between the other two approaches and have the same interpretation as before. However, the interaction effect is slightly larger resulting in a significant effect (*t* = 2.118, *p* = 0.039). This result shows the T+S group has a benefit over placebo. The slope variance of study period is equal to 18.01. This means that there is substantial variation in the effect of study period across patients, after differences due to treatment group have been considered (cross-level interaction). For placebo patients, the average increase from BLE to ATP is 2.97, and the random slope variation shows that approximately 67% of the placebo patients have a predicted change from BLE to ATP lying within the predictive interval $2.97\;-\;\sqrt{18.01}\;=\;-\;1.28$ and $2.97\;+\;\sqrt{18.01}\;=\;-\;7.21$. For the T+S patients, the average increase from BLE to ATP is 2.97 + 2.88 = 5.85, and the random slope variation shows that approximately 67% of the T+S patients have a predicted increase from BLE to ATP lying within the predictive interval $5.85\;-\;\sqrt{18.01}\;=\;1.60$ and $5.85\;+\;\sqrt{18.01}\;=\;10.09$. The estimated intercept variance of this model is 17.17 leading to 67% predictive intervals ranging from 9.04 to 17.33 for the placebo patients and 7.28 to 15.57 for the T+S patients.

The inclusion of a random slope for study period in the model leads to more error around the fixed effects, because the variability in individual slopes is taken into account when estimating the average effect. This leads to an estimate of which we are less certain and thus more error. One could argue not to include the random slope, because the main interest in drug development research is focused on the fixed effects. However, not including random slope variation when it clearly exists leads to downward biased standard errors for the fixed effects [38]. This is undesirable, because it results in estimated fixed effects that have too narrow confidence intervals. Furthermore, an underestimated standard error will also result in a biased p-value and may therefore lead to erroneous conclusions. It is therefore recommended to include a random slope if there is slope variation, even when one is interested in the fixed effects only (provided that there are sufficient second-level units, see Bell et al. [38]). As outlined in the Introduction Section, the inclusion of a random slope enables the examination of varying drug effects across patients which we believe could offer a valuable contribution to the evaluation of clinical trial results.

**Fig 1:**
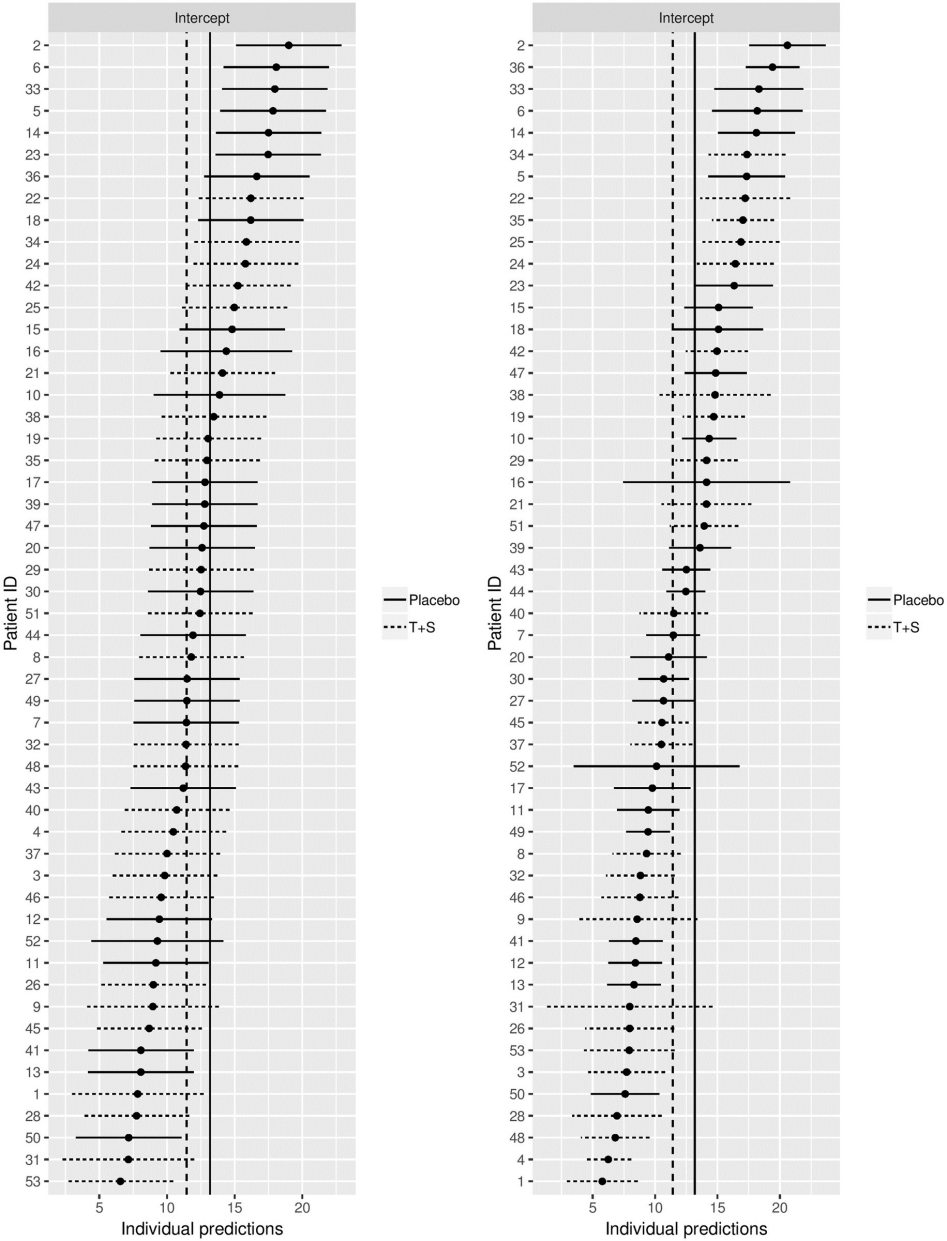
Caterpillar plots of the individual predicted intercepts and their 95% confidence intervals. The left plot shows the predicted intercepts for Model (1) and the right plot for Model (2). The vertical lines present the fixed intercepts for both treatment groups.

Let us take a closer look at these varying drug effects. Although they appear in the model equations for *Y*_*ei*_, the individual *u*_0*i*_ and *u*_1*i*_ values are not estimated directly. The individual *u*_0*i*_ and *u*_1*i*_ values represent the difference between the fixed intercept and the predicted intercept and the fixed slope and predicted slope for patient *i*. When using the lme4 package, the individual predicted intercepts and slopes can be obtained. In Fig 1, two so-called caterpillar plots are presented where each individual predicted intercept is plotted together with the individual 95% confidence intervals. The confidence intervals reflect the accuracy of the individual predicted intercepts. The left plot shows the predicted intercepts of Model (1) and the right plot shows the predicted intercepts of Model (2). In the right plot, there is much more variation in the widths of the confidence intervals compared to the left plot where almost all confidence intervals have equal width. This is because in Model (2) the underlying standard error of each individual predicted average is taken into account. Patients reporting more events during the BLE period have a smaller standard error and thus a smaller confidence interval which indicates a more accurate prediction. For example, patient ID number 44 reported 14 events during the BLE period resulting in a small confidence interval width and an accurate prediction when applying Model (2) to the individual event data (right plot). The opposite holds for patient ID number 33 who reported only 2 events resulting in a much wider confidence interval and a less accurate estimate compared to patient ID number 44. However, the confidence interval of patient ID number 33 is a bit smaller than the confidence interval of patient ID number 35, who reported 5 events during the BLE period. This difference is because the variance of the scores of patient ID number 35 (variance of 59.7) is larger than the variance of the scores of patient ID number 33 (variance of 2.0). This additional information is ignored when predicting the individual intercepts in estimating Model (1) on the aggregated data where these three patients have confidence intervals of equal width (left plot).

**Fig 2:**
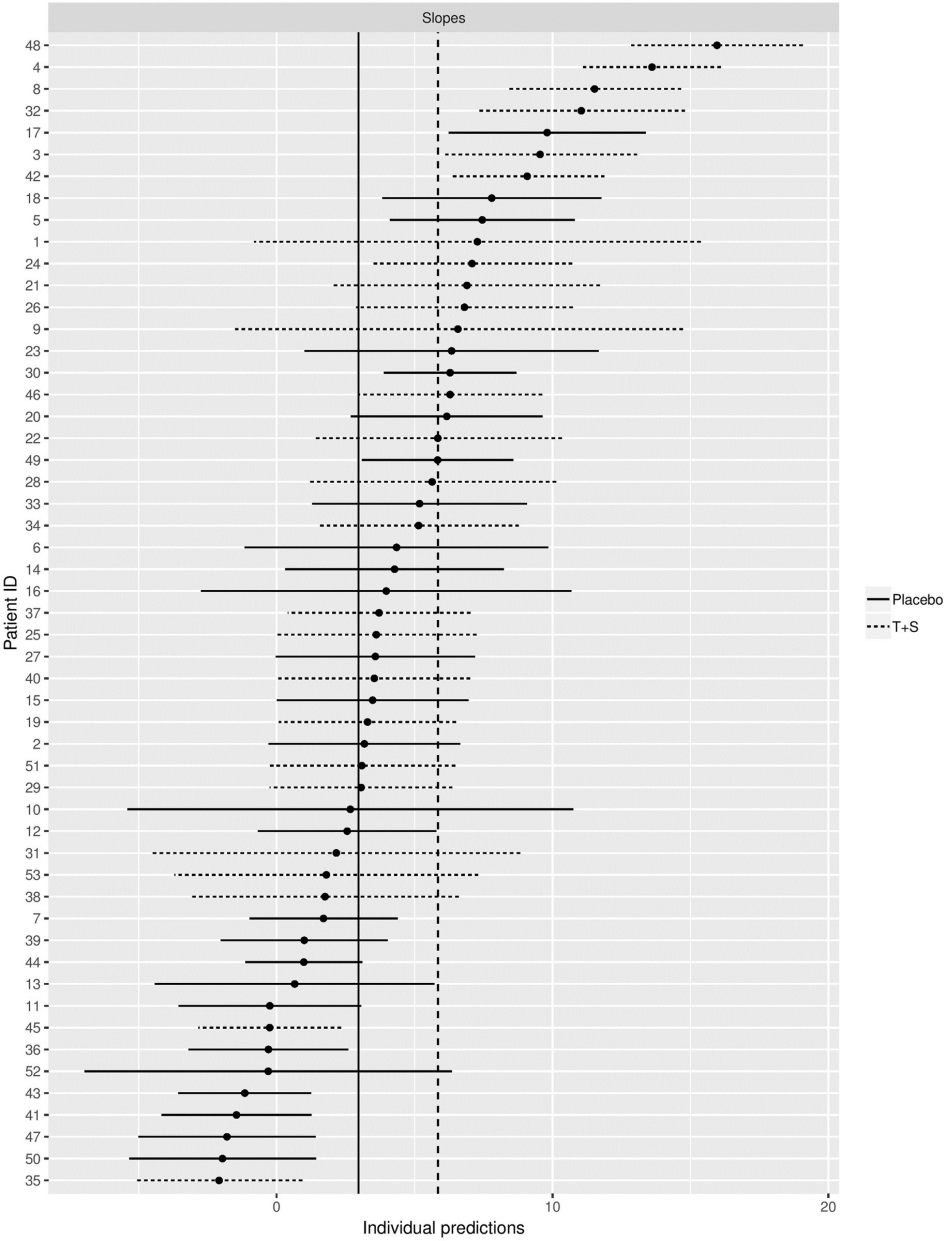
Caterpillar plot of the individual predicted slopes of study period and their 95% confidence intervals for Model (2). The vertical lines present the fixed slope estimates for both treatment groups.

An additional advantage of applying Model (2) to the individual event data is that a random slope can be estimated. Therefore, we can also obtain individual slope predictions along with their confidence intervals, which are shown in Fig 2. Also here, the different underlying standard errors of each individual predicted sexual functioning score during the ATP are taken into account. In this figure, the four patients showing the largest change from baseline in sexual functioning were randomized to the T+S condition.

In sum, these results show that there is a benefit of T+S compared to placebo and that there is a large variability over individuals in the effect of the drug.

In addition to the intercept and slope variances, Model (2) also estimates a correlation (covariance) between the random intercept and the random slope. This correlation is equal to -0.25 and shows that patients with lower sexual function scores at baseline have a steeper increase over time.

The multilevel analysis of the individual events was repeated with patient-level covariates age (mean = 43.1, Standard Deviation (SD) = 11.24), Body Mass Index (BMI) score (mean = 26.7, SD = 7.27), and menopausal status (71.7% premenopausal, 28.3% postmenopausal). BMI and age were grand-mean centered by subtracting the grand-mean from all individual values such that the average age and BMI became zero. With the inclusion of the fixed effects of these covariates, the random intercept *β*_0*i*_ can be rewritten as *γ*_00_ + *γ*_01_*Z*_1*i*_ + *γ*_02_*Z*_2*i*_ + *γ*_03_*Z*_3*i*_ + *γ*_04_*Z*_4*i*_ + *u*_0*i*_, where *Z*_1*i*_,…,*Z*_4*i*_ are the patient-level predictors treatment group, age, BMI, and menopausal status. The results of this model are presented in the last column of Table 2. All three covariates have a small non-significant effect and the parameters only change slightly. Because the previous model is nested within this model by setting the regression coefficients for age, BMI and menopausal status equal to zero, we can compare both models using the deviance difference test. The result of the deviance difference test revealed the model without the covariates did not fit worse (*χ*^2^ = 0.85, *df* = 3, *p* = 0.837). These results indicate the same conclusions could be drawn after controlling for these three additional covariates.

### Trend analyses: The learning effect

Because the additional covariates age, BMI, and menopausal status did not improve the model fit, trend analyses were conducted without these covariates. The results of the model with a fixed linear effect only are listed in the first column of Table 3. The fixed linear effect is only 0.06 and suggests an insignificant positive learning effect over time in both the BLE period and the ATP. The extension of this model with a random slope for the linear term (*β*_2*i*_ = *γ*_20_ + *u*_2*i*_) did not improve the model according to a deviance difference test (*χ*^2^ = 2.17, *df* = 3, *p* = 0.538). The random slope variance for the linear term was even close to zero. This means that the linear learning effect does not vary across patients (estimates are not shown).

**Table 3:** Estimated parameters for the trend models.

Model	Model with linear trend		Model with linear and quadratic trend	
Fixed part (multilevel notation)	Coef (SE)	*p*-value	Coef (SE)	*p*-value
Intercept (*γ*_00_)	13.04 (0.88)		12.81 (0.89)	
Study period (*γ*_10_)	2.87 (0.94)	0.004	2.85 (0.94)	0.004
Treatment group (*γ*_01_)	-1.71 (1.26)	0.180	-1.65 (1.26)	0.197
Study period×Treatment group (*γ*_11_)	2.84 (1.35)	0.041	2.79 (1.35)	0.044
Event Count (*γ*_20_)	0.06 (0.04)	0.134	0.23 (0.10)	0.024
(Event Count)^2^(*γ*_30_)			-0.014 (0.01)	0.073
**Random part**				
$\sigma_{\varepsilon }^{2}$	9.36		9.29	
$\sigma_{u0}^{2}$	17.39		17.65	
$\sigma_{u1}^{2}$	17.76		17.70	
*r*(*u*_0_,*u*_1_)	-0.25		-0.25	
**Deviance**	3409.2		3406.0	
**AIC**	3427.2		3426.0	

Abbreviations: Coef = Coefficient, SE = Standard error, AIC = Akaike Information Criterion

The results of the model with a fixed linear and quadratic term are listed in the second column of Table 3. The fixed quadratic effect is negative (-0.014, *t* = -1.799, *p* = 0.073). This quadratic effect is not significant which implies there is no effect of event count. However, when comparing the AIC’s of the models on the individual event data (see Tables 2 and 3), the model with the cross-level interaction between study period and treatment group and the fixed linear and quadratic term for event count has the best model fit, as this model has the lowest AIC value (see second column Table 3). Furthermore, because the main goal of this paper was to illustrate the additional modeling possibilities that become available when considering the events as nested observations, we left the fixed linear effect and fixed quadratic effect of event count in the model. The negative value of the quadratic effect means that the learning effect attenuates over time for both periods. When taking the first derivative with respect to *T* (0.23-0.028*T*) and solving for *T* by setting the first derivative equal to zero, it shows that after approximately eight events, the increasing learning effect starts to decline. In 23% of the observed study periods, the total number of events exceeds eight. This means that in all other cases, no decline in learning occurs. No random effect for the quadratic term was included as such a model did not converge. We also investigated whether the learning effect was different between the BLE period and ATP by including the interaction effect between the linear term and study period and the interaction effect between the quadratic term and study period; however, the deviance difference test was not significant (*χ*^2^ = 1.39, *df* = 2, *p* = 0.500).

### Adding the patient-means of study period and event count

In this section, we discuss the results of adding the patient means of study period and event count to investigate whether the within and between effects of these variables are equal. We started this analysis by adding the patient means of study period. These results are presented in the first column of Table 4. Compared to the results listed in the second column of Table 3, the fixed and random effect estimates of the parameters present in both models do not differ much, but the interpretation has changed. In the previous model, the fixed effect of study period is equal to 2.85 and the cross-level interaction is equal to 2.79 (see Table 3). These fixed effects consist of a within-patient effect and a between-patients effect and could be interpreted as the change from baseline for placebo patients and the additional change for T+S patients. After adding the patient means of study period, the fixed effects of study period and the cross-level interaction are estimated at 2.71 and 2.78 and can now be interpreted as the within-patient effect of study period controlling for the patient means of study period. In other words, within patients receiving placebo, the average change from baseline in sexual functioning is 2.71 and within patients receiving T+S, this change is equal 2.71 + 2.78 = 5.49. The fixed effect of the study period means (*γ*_02_) and the fixed effect of the interaction between the study period means and treatment group (*γ*_03_) are 4.37 and 0.94 respectively and represent the difference between the within-patient effects and between-patient effects. These effects, however, are both not statistically significant from zero which indicates that the within and between effects are equal. It is therefore permissible to remove the patient means from the model. Also, the addition of both parameters did not improve the model fit as the AIC (3427.0) is larger compared to the AIC of the model presented in the second column of Table 3 (3426.0).

**Table 4:** Estimated parameters for multilevel models including the patient means of study period and event count.

Model	Model with Study Period means		Model with Event count means	
Fixed part (multilevel notation)	Coef (SE)	*p*-value	Coef (SE)	*p*-value
Intercept (*γ*_00_)	13.06 (0.89)		12.65 (1.03)	
Study period (*γ*_10_)	2.71 (0.95)	0.007	2.84 (0.94)	0.004
Treatment group (*γ*_01_)	-1.76 (1.25)	0.166	-1.76 (1.28)	0.175
Study period×Treatment group (*γ*_11_)	2.78 (1.37)	0.048	2.80 (1.35)	0.044
Event Count (*γ*_20_)	0.23 (0.10)	0.025	0.23 (0.10)	0.023
(Event Count)^2^(*γ*_30_)	-0.014 (0.01)	0.073	-0.014 (0.01)	0.070
Study period means (*γ*_02_)	4.37 (3.73)	0.247		
Study period means×Treatment group (*γ*_03_)	0.94 (5.50)	0.865		
Event Count means (*γ*_04_)			-0.16 (0.34)	0.635
(Event Count means)^2^(*γ*_05_)			0.017 (0.16)	0.913
**Random part**				
$\sigma_{\varepsilon }^{2}$	9.29		9.29	
$\sigma_{u0}^{2}$	16.41		17.39	
$\sigma_{u1}^{2}$	17.99		17.77	
*r*(*u*_0_, *u*_1_)	-0.26		-0.24	
**Deviance**	3403.0		3405.7	
**AIC**	3427.0		3429.7	

Abbreviations: Coef = Coefficient, SE = Standard error, AIC = Akaike Information Criterion

Note that the estimated value of 4.37 of the fixed effect of the patient means seems large at first notice. However, this reflects the change in the dependent variable after one unit increase in the patient mean of study period. Because study period is a dichotomous predictor (0 = BLE, 1 = ATP) a unit increase in the average indicates the difference between reporting no events during the ATP (patient-mean is equal to zero) and reporting only events during the ATP (patient-mean is equal to 1). Given that the average number of reported events across patients is 11.79, we can approximate the mean change in sexual functioning per additional ATP event as 4.37 / 11.79 = 0.37. The same conversion can be applied to the estimate of *γ*_03_.

Subsequently, the patients means of the event count variable were added to the model (see second column of Table 4). Also here, the parameter estimates do not differ much compared to the previous models and the fixed effects of the event count means and the squared event count means are not significantly different from zero. These analyses lead to the same conclusion regarding the difference between the within-patient and between-patient effects of these variables and both parameters can be deleted from the model. Also, the AIC of this model shows that the model fits worse compared to the model presented in the second column of Table 3.

## Discussion

This paper presented different applications of multilevel modeling to on-demand medication intake data with a focus on an on-demand treatment for FSIAD. Because this kind of data is often aggregated into means and subsequently analyzed using an ANOVA or multilevel approach, our study started with a comparison between these two approaches using these aggregated data. Comparisons between these two methods on (balanced) repeated measurement data were performed and described in the literature in several other research areas, for example asthma in children [39], fetal heart rate variability [40], psychosomatic medicine [41], and intra-articular fractures [42]. It can be proven that, when only a random intercept is included, both models produce equivalent results when complete data are provided. However, when there are missing observations, the BWS ANOVA uses listwise deletion to handle the missing observations whereas in the multilevel approach, observations with missing values on the dependent variable are allowed, assuming the missingness is Missing at Random.

A problem that arises when aggregating the data into means is specifically attributable to the nature of on-demand medication data, where events of varying frequencies between patients are observed. Patient means have different standard errors attached to them when the variance of the underlying event scores differs between patients and/or when the number of events per patient differs. This additional information is ignored when aggregating the data into means before the analysis.

Therefore, in this paper, a third multilevel approach was discussed that solves the abovementioned problem by specifying the individual events as first-level observations nested within patients. Although the main interest in drug development research is focused on the fixed drug effect, we pointed out that an additional advantage of this multilevel modeling approach is that it enables the examination of varying drug effects by estimating a random slope for this drug effect. We showed by calculating predictive intervals around the random intercept and slope using the estimated variance components how to give fixed and random effects meaningful interpretations. We further illustrated how the individual standard errors that are attached to the individual patient means are considered when predicting individual drug effects. These individual effects can offer a valuable contribution to the evaluation of clinical trial results because they give an insight in which patient benefits the most.

When we compare the multilevel analysis on the aggregated scores with the multilevel analysis on the individual events, an interesting question is whether and when one of the two analyses has more power for testing the fixed effects than the other. On the one hand, the multilevel analysis on the individual events makes use of more data and this could lead to more power. However, it is likely that the outcome variable (sexual functioning) for each event in the analysis on the individual events is measured with error, and that the events within a patient are correlated. Then, by aggregating the event scores into means, the resulting average outcome variable per patient will have a smaller measurement error than the outcome per event. Furthermore, aggregating the data will also lead to less variability. So, using the individual events leads to more data, but it also means that the outcome has more error and variability than the aggregated score. These two tendencies work in opposite directions concerning the statistical power. Monte Carlo simulations could be a tool to investigate under what circumstances an analysis using events is more powerful than an analysis using averages. However, we consider this to be beyond the scope of the paper and see this as future work.

This paper also focused on exploring some additional modeling possibilities that can only be performed when employing the multilevel analysis on individual events. Linear and quadratic trends were modeled through the inclusion of a variable counting the events. An individual change over time occurs continuously. Analyzing time effects in a multilevel model involving only two (aggregated) scores is not the most optimal method of analyzing such change [43]. In longitudinal research, multiple measurements per individual are preferred to reliably estimate continuous change. The advantage of using individual events is that these events can be viewed as a collection of multiple data measurements; thus, the advocated multilevel approach is suitable for analyzing change, or trends over time. In this paper, a linear and a quadratic trend were included to study the learning effect of sexual behavior over time. Although the relevance of the linear and quadratic effect for event count can be questioned considering the non-significant p-values, the negative quadratic effect showed that the learning effect declines over time. These trend analyses provided interesting results that might be left unnoticed when conducting analyses on the aggregated scores.

Finally, we explained the problem of mixed between and within effects in first-level variables, which is an important issue in multilevel modeling. By adding the patient means of the included first-level variables, we demonstrated a way to test whether within and between effects differ. Another method to solve the mixing-up of between and within effects is to remove the between-patient effect from the within-patient effect by subtracting the patient means from the corresponding scores. This procedure is known as patient-mean centering and leads to unbiased within-patient effects [1, 2, 44]. Because patient-mean centering discards all information concerning differences between patients, this information is often restored by adding the patient means at the second level of the multilevel model. This approach is preferred when interest is focused on both the within and between effects because patient-mean centering a first-level variable and adding the patient means as a predictor clearly separates the pure within-patient effect from the pure between-patient effect. However, this method changes the entire regression model and complicates the interpretation of the parameters.

In this paper, we argued why and demonstrated how on-demand medication data should be analyzed with multilevel analyses using the individual observed events as separate nested observations. Using the individual events rather than aggregating over the events takes into account the different standard errors associated with the patient averages, enables examination of varying drug effects and allows the inclusion of relevant information available at the event-level.

## Supporting information

S1 Appendix R-code used to generate the results. (PDF)

## References

[1] Raudenbush SW, Bryk AS. Hierarchical linear models: Applications and data analysis methods, 2nd Edn Thousand Oaks, CA: Sage Publications; 2002.

[2] Hox J, Moerbeek M, Van De Schoot R. Multilevel analysis: Techniques and applications, 3rd Edn New York, NJ: Taylor & Francis Group; 2018.

[3] Goldstein H. Multilevel statistical models, 4th edition Chichester: Wiley; 2010.

[4] Snijders TA, Bosker RJ. Multilevel Analysis: An introduction to basic and advanced multilevel modeling, 2 Edn London: Sage Publishers; 2012.

[5] Singer JD, Willett JB. Applied longitudinal data analysis: Modeling change and event occurrence. Oxford university press; 2003 10.1093/acprof:oso/9780195152968.001.0001

[6] Goldstein I, Lue TF, Padma-Nathan H, Rosen RC, Steers WD, Wicker PA. Oral sildenafil in the treatment of erectile dysfunction. N Engl J Med. 1998;338:1397–1404. 10.1056/NEJM199805143382001 9580646

[7] Padma-Nathan H, McMurray J, Pullman W, Whitaker J, Saoud J, Ferguson K, et al On-demand IC351 (Cialis (TM)) enhances erectile function in patients with erectile dysfunction. Int J Impot Res. 2001;13:2–9. 10.1038/sj.ijir.3900631 11313831

[8] Skoumal R, Chen J, Kula K, Breza J, Calomfirescu N, Basson BR, et al Efficacy and treatment satisfaction with on-demand Tadalafil in men with erectile dysfunction. Eur Urol. 2004;46:362–369. 10.1016/j.eururo.2004.04.026 15306109

[9] Buvat J, Tesfaye F, Rothman M, Rivas DA, Giuliano F. Dapoxetine for the treatment of premature ejaculation: results from a randomized, double-blind, placebo-controlled phase 3 trial in 22 countries. Eur Urol. 2009;55:957–968. 10.1016/j.eururo.2009.01.025 19195772

[10] Bar-Or D, Salottolo KM, Orlando A, Winkler JV, Group TOS, et al A randomized double-blind, placebo-controlled multicenter study to evaluate the efficacy and safety of two doses of the tramadol orally disintegrating tablet for the treatment of premature ejaculation within less than 2 minutes. Eur Urol. 2012;61:736–743. 10.1016/j.eururo.2011.08.039 21889833

[11] Clayton AH, Althof SE, Kingsberg S, DeRogatis LR, Kroll R, Goldstein I, et al Bremelanotide for female sexual dysfunctions in premenopausal women: a randomized, placebo-controlled dose-finding trial. Women’s Health. 2016;12:325–337. 10.2217/whe-2016-0018 27181790 PMC5384512

[12] Tuiten A, Van Rooij K, Bloemers J, Eisenegger C, van Honk J, Kessels R, et al Efficacy and safety of on-demand use of 2 treatments designed for different etiologies of female sexual interest/arousal disorder: 3 randomized clinical trials. J Sex Med. 2018;15:201–216. 10.1016/j.jsxm.2017.11.226 29289554

[13] Manco-Johnson M, Kempton C, Reding M, Lissitchkov T, Goranov S, Gercheva L, et al Randomized, controlled, parallel-group trial of routine prophylaxis vs. on-demand treatment with sucrose-formulated recombinant factor VIII in adults with severe hemophilia A (SPINART). J Thromb Haemost. 2013;11:1119–1127. 10.1111/jth.12202 23528101

[14] Szarka LA, Camilleri M, Burton D, Fox JC, McKinzie S, Stanislav T, et al Efficacy of on-demand asimadoline, a peripheral *κ*-opioid agonist, in females with irritable bowel syndrome. Clin Gastroenterol Hepatol. 2007;5:1268–1275. 10.1016/j.cgh.2007.07.011 17900994 PMC2128734

[15] Galmiche J, Shi G, Simon B, Casset-Semanaz F, Slama A. On-demand treatment of gastro-oesophageal reflux symptoms: a comparison of ranitidine 75 mg with cimetidine 200 mg or placebo. Aliment Pharmacol Therapeut. 1998;12:909–917. 10.1046/j.1365-2036.1998.00384.x 9768535

[16] Johnsson F, Moum B, Vilien M, Grove O, Simren M, Thoring M. On-demand treatment in patients with oesophagitis and reflux symptoms: comparison of lansoprazole and omeprazole. Scand J Gastroenterol. 2002;37:642–647. 10.1080/00365520212499 12126240

[17] Kent DM, Steyerberg E, Van Klaveren D. Personalized evidence based medicine: predictive approaches to heterogeneous treatment effects. BMJ. 2018;363:k4245 10.1136/bmj.k4245 30530757 PMC6889830

[18] Cnaan A, Laird N, Slasor P. Using the general linear mixed model to analyse unbalanced repeated measures and longitudinal data. Stat Med. 1997;16:2349–2380. 10.1002/(SICI)1097-0258(19971030)16:20<2349::AID-SIM667>3.0.CO;2-E 9351170

[19] Ellenberg SS. Discussion: Is the FDA in need of a major change in the way it regulates? Biostatistics. 2017;18:414–416. 10.1093/biostatistics/kxx022 28633289

[20] Brown H, Prescott R. Applied mixed models in medicine, 3rd Edn Chichester: John Wiley & Sons; 2015.

[21] Gosho M, Maruo K. Effect of heteroscedasticity between treatment groups on mixed-effects models for repeated measures. Pharm Stat. 2018;17:578–592. 10.1002/pst.1872 29978944

[22] Mallinckrod CH, Lane PW, Schnell D, Peng Y, Mancuso JP. Recommendations for the primary analysis of continuous endpoints in longitudinal clinical trials. Drug Inf J. 2008;42:303–319. 10.1177/009286150804200402

[23] Mallinckrodt CH, Watkin JG, Molenberghs G, Carroll RJ. Choice of the primary analysis in longitudinal clinical trials. Pharm Stat. 2004;3:161–169. 10.1002/pst.124

[24] Van Nes Y, Bloemers J, Van Der Heijden P, Van Rooij K, Gerritsen J, Kessels R, et al The Sexual Event Diary (SED): Development and Validation of a Standardized Questionnaire for Assessing Female Sexual Functioning During Discrete Sexual Events. J Sex Med. 2017;14:1438–1450. 10.1016/j.jsxm.2017.09.008 28993148

[25] Pfaus JG, Erickson KA, Talianakis S. Somatosensory conditioning of sexual arousal and copulatory behavior in the male rat: A model of fetish development. Physiol Behav. 2013;122:1–7. 10.1016/j.physbeh.2013.08.005 23954746

[26] Palace EM. A Cognitive-Physiological process model of sexual arousal and response. Clin Psychol-Sci Pr. 1995;2:370–384. 10.1111/j.1468-2850.1995.tb00049.x

[27] Kreft IG, Kreft I, de Leeuw J. Introducing multilevel modeling. Thousand Oaks, CA: Sage; 1998 10.4135/9781849209366

[28] Maxwell SE, Delaney HD. Designing experiments and analyzing data: A model comparison perspective. vol. 1 Psychology Press; 2004.

[29] Hoffman L. Longitudinal analysis: Modeling within-person fluctuation and change. Routledge; 2015.

[30] R Core Team. R: A Language and Environment for Statistical Computing; 2019 Available from: https://www.R-project.org/.

[31] Bates D, Mächler M, Bolker B, Walker S. Fitting linear mixed-effects models using lme4. J Stat Softw. 2015;67 10.18637/jss.v067.i01

[32] Kuznetsova A, Brockhoff PB, Christensen RHB. lmerTest package: tests in linear mixed effects models. J Stat Softw. 2017;82 10.18637/jss.v082.i13

[33] McNeish DM, Stapleton LM. The effect of small sample size on two-level model estimates: A review and illustration. Educ Psychol Rev. 2016;28:295–314. 10.1007/s10648-014-9287-x

[34] Satterthwaite FE. An approximate distribution of estimates of variance components. Biometrics bulletin. 1946;2:110–114. 10.2307/3002019 20287815

[35] Manor O, Zucker DM. Small sample inference for the fixed effects in the mixed linear model. Comput Stat Data An. 2004;46:801–817. 10.1016/j.csda.2003.10.005

[36] Berkhof J, Snijders TA. Variance component testing in multilevel models. J Educ Behav Stat. 2001;26:133–152. 10.3102/10769986026002133

[37] Akaike H. Factor analysis and the AIC. Psychometrika. 1987;52:317–332. 10.1007/BF02294359

[38] Bell A, Fairbrother M, Jones K. Fixed and random effects models: making an informed choice. Qual Quant. 2019;53:1051–1074. 10.1007/s11135-018-0802-x

[39] Omar R, Wright E, Turner R, Thompson S. Analysing repeated measurements data: a practical comparison of methods. Stat Med. 1999;18:1587–1603. 10.1002/(SICI)1097-0258(19990715)18:13<1587::AID-SIM141>3.0.CO;2-Z 10407231

[40] Krueger C, Tian L. A comparison of the general linear mixed model and repeated measures ANOVA using a dataset with multiple missing data points. Biol Res Nurs. 2004;6:151–157. 15388912 10.1177/1099800404267682

[41] Blackwell E, de Leon CFM, Miller GE. Applying mixed regression models to the analysis of repeated-measures data in psychosomatic medicine. Psychosom Med. 2006;68:870–878. 10.1097/01.psy.0000239144.91689.ca 17079709

[42] Kwok OM, Underhill AT, Berry JW, Luo W, Elliott TR, Yoon M. Analyzing longitudinal data with multilevel models: An example with individuals living with lower extremity intra-articular fractures. Rehabil Psychol. 2008;53:370 10.1037/a0012765 19649151 PMC2613314

[43] Willett JB, Singer JD, Martin NC. The design and analysis of longitudinal studies of development and psychopathology in context: Statistical models and methodological recommendations. Dev Psychopathol. 1998;10:395–426. 10.1017/S0954579498001667 9635230

[44] Enders CK, Tofighi D. Centering predictor variables in cross-sectional multilevel models: a new look at an old issue. Psychol Methods. 2007;12:121–38. 10.1037/1082-989X.12.2.121 17563168

