# Correction: Multilevel analyses of on-demand medication data, with an application to the treatment of Female Sexual Interest/Arousal Disorder

**DOI:** 10.1371/journal.pone.0224708

**Published:** 2019-10-28

**Authors:** The PLOS ONE Staff

In the Model for analyzing individual events subsection of the Methods, there are errors in third and fourth equation of Model (2). Please view the complete, correct equations here:

$$\beta_{1i}=\gamma_{10}+\gamma_{11}Z_{i}+u_{1i}$$

$$\mathrm{Single}\;\mathrm{equation}:Y_{ei}=\gamma_{00}+\gamma_{10}X_{ei}+\gamma_{01}Z_{i}+\gamma_{11}Z_{i}X_{ei}+u_{1i}X_{ei}+u_{0i}+\varepsilon_{ei}$$

The publisher apologizes for the error.
