# Supplementary material for: Multilevel analyses of on-demand medication data, with an application to the treatment of Female Sexual Interest/Arousal Disorder
Source: PLoS One. 2019 Aug 15;14(8):e0221063. doi: 10.1371/journal.pone.0221063 (PMC6695215; doi:10.1371/journal.pone.0221063)
Supplement: S1 Appendix — (PDF) [file pone.0221063.s001.pdf]

S1 Appendix

R-code used to generate the results

```
#Datasets used can be found at
https://github.com/ebkesselsr/Multilevel_fsiad

#Load packages (if packages need to be installed first, do:
install.packages("packagename")

require(psych)
require(lme4)
require(haven)
require(lmerTest)
require(plyr)
require(dplyr)
require(ggplot2)


#########################################################
#R-code for Factor analysis on complete data (Section 2)#
#########################################################

#Load data
data.factor <- read_spss("EB82 Multilevel Analysis Full Data.sav")
data.factor <- as.data.frame(data.factor)

items       <- data.factor[,c(5:9)]
covmat      <- cov(items, use="complete.obs")

#Exploratory factor analysis using a parallel analysis with WLS estimator
fit0        <- fa.parallel(x=items, fm="wls", fa="fa", cor="poly")
fit0

#Factor analysis extracting 1 factor using WLS estimator
fit0.1      <- fa(items, nfactors=1, n.obs=1495, covar=TRUE, fm="wls",
                 cor="poly")
fit0.1

#Calculate the Cronbach's alpha
CA          <- psych::alpha(items)$total$std.alpha

#Remove objects
rm(list = ls())


#################################################
# R-code for analyses performed in Section 3.1.1#
#################################################

#Models Section 3.1

#Load data
data.agg     <- read_spss("EB82 Multilevel Analysis Section 3.1.1.sav")

#Delete patients with missing data for analysis analogous to BWS ANOVA

data2 <- ddply(data.agg, "id",
               function(df)if(any(is.na(df[, 3]))) NULL else df)
```

```r
fit1  <- lmer(SexualFunction~StudyPeriod*Treatment +
              (1|id), REML=FALSE ,data=data2)
summary(fit1)

#67% predictive interval random intercept placebo and T+S patients
lo.p <- fixef(fit1)[1] - sqrt(13.47)
hi.p <- fixef(fit1)[1] + sqrt(13.47)

lo.t <- (fixef(fit1)[1] + fixef(fit1)[3]) - sqrt(13.47)
hi.t <- (fixef(fit1)[1] + fixef(fit1)[3]) + sqrt(13.47)

#ML on aggregated scores including a random slope (use all observed
patients, including the ones with missings)
fit2  <- lmer(SexualFunction~StudyPeriod*Treatment +
               (1 + StudyPeriod|id),
              data=data.agg, REML=FALSE, na.action=na.omit)

#Model estimation runs into errors as the variance-covariance matrix of the
random effects is unidentifiable

#Fit a model with only a random intercept

fit2a  <- lmer(SexualFunction~StudyPeriod*Treatment +
               (1|id), REML=FALSE, data=data.agg, na.action=na.omit)
summary(fit2a)

#Figure 2 (left plot)
#Caterpillar plot random intercepts Model(2)
var1            <- attr(ranef(fit2a, condVar=T)[[1]], "postVar")
inter           <- var1
se1             <- unlist(lapply(1, function(i) sqrt(inter[i, i, ])))
pDf.var1        <- data.frame(y=ranef(fit2a, condVar=T)[[1]][,1],
                          ci=1.96*se1,
                          id=factor(rownames(ranef(fit2a,
                          condVar=T)[[1]]),
                          levels=rownames(ranef(fit2a,
                          condVar=T)[[1]])),
                          ind="Intercept")

fixint_p        <- fixef(fit2a)[1]
fixint_t        <- fixef(fit2a)[1] + fixef(fit2a)[3]

trt.group       <- as.data.frame(distinct(data.agg[,c(1,2)]))
trt.group$id    <- as.factor(trt.group$id)

trt.group       <- cbind(trt.group, fixint_p, fixint_t)

pDf1            <- full_join(pDf.var1, trt.group, by="id")
pDf1$trt.label  <- ifelse(pDf1$Treatment==0, "Placebo", "T+S")

for (i in 1:nrow(pDf1)){
    if(pDf1$Treatment[i]==0) {pDf1$y2[i] = pDf1$y[i] + pDf1$fixint_p[i]
    } else {pDf1$y2[i] = pDf1$y[i] + pDf1$fixint_t[i]
      }
}

p1  <- ggplot(pDf1, aes(reorder(id, y2),y2)) + coord_flip()
p1  <- p1 + facet_wrap(~ ind)
p1  <- p1 + xlab("Patient ID") + ylab("Individual predictions")
p1  <- p1 + theme(legend.title=element_blank())
#p1  <- p1 + theme(legend.position="none")
```

```
#Commented out to show legend
p1  <- p1 + geom_hline(yintercept=c(fixint_p, fixint_t), linetype=c(1, 2))
p1  <- p1 + geom_errorbar(aes(ymin=y2-ci, ymax=y2+ci,
                          linetype=factor(trt.label)), width=0)
p1  <- p1 + geom_point(aes())


##################################################
# R-code for analyses performed in Section 3.1.2#
##################################################

#Read data
data     <- read_spss("EB82 Multilevel Analysis Section 3.1.2 - 3.2
                       3.3.sav")

#ML model on individual events.

fit1  <- lmer(SexualFunction~ StudyPeriod*Treatment+
              (1 + StudyPeriod|id), data=data, REML=FALSE,
               na.action=na.omit)
summary(fit1)

#67% predictive interval slope placebo and T+S patients
lo.p.s <- fixef(fit1)[2] - sqrt(18.01)
hi.p.s <- fixef(fit1)[2] + sqrt(18.01)

lo.t.s <- (fixef(fit1)[2]+fixef(fit1)[4]) - sqrt(18.01)
hi.t.s <- (fixef(fit1)[2]+fixef(fit1)[4]) + sqrt(18.01)

#67% predictive interval intercept for placebo and T+S patients
lo.p.i <- fixef(fit1)[1] - sqrt(17.17)
hi.p.i <- fixef(fit1)[1] + sqrt(17.17)

lo.t.i <- (fixef(fit1)[1]+fixef(fit1)[3]) - sqrt(17.17)
hi.t.i <- (fixef(fit1)[1]+fixef(fit1)[3]) + sqrt(17.17)

#Figure 2 (right plot) and Figure 3
#Caterpillar plot random intercepts Model(3)

f <- function(x) {
    pv   <- attr(x, "postVar")
    cols <- 1:(dim(pv)[1])
    se   <- unlist(lapply(cols, function(i) sqrt(pv[i, i, ])))
    ord  <- unlist(lapply(x, order)) + rep((0:(ncol(x) - 1)) * nrow(x),
                 each=nrow(x))
    pDf  <- data.frame(y=unlist(x)[ord],
                       ci=1.96*se[ord],
                       id=factor(rep(rownames(x), ncol(x))[ord],
                       levels=rownames(x)[ord]),
                       ind=gl(ncol(x), nrow(x), labels=c("Intercept",
                       "Slopes")))
    return(pDf)
}
pDf2   <- lapply(ranef(fit1, condVar=T), f)
pDf2   <- pDf2$id

pDf.i  <- subset(pDf2, ind=="Intercept")

fixint_p        <- fixef(fit1)[1]
fixint_t        <- fixef(fit1)[1] + fixef(fit1)[3]
```

```r
trt.group       <- as.data.frame(distinct(data[,c(1,2)]))
trt.group$id    <- as.factor(trt.group$id)
trt.group       <- cbind(trt.group, fixint_p, fixint_t)

pDf.i           <- full_join(pDf.i, trt.group, by="id")
pDf.i$trt.label <- ifelse(pDf.i$Treatment==0, "Placebo", "T+S")

for (i in 1:nrow(pDf.i)){
    if(pDf.i$Treatment[i]==0) {pDf.i$y2[i] = pDf.i$y[i] + pDf.i$fixint_p[i]
    } else {pDf.i$y2[i] = pDf.i$y[i] + pDf.i$fixint_t[i]
      }
}

p2  <- ggplot(pDf.i, aes(reorder(id, y2),y2)) + coord_flip()
p2  <- p2 + facet_wrap(~ ind)
p2  <- p2 + xlab("Patient ID") + ylab("Individual predictions")
p2  <- p2 + theme(legend.title=element_blank())
p2  <- p2 + geom_hline(yintercept=c(fixint_p, fixint_t), linetype=c(1, 2))
p2  <- p2 + geom_errorbar(aes(ymin=y2-ci, ymax=y2+ci,
            linetype=factor(trt.label)), width=0)
p2  <- p2 + geom_point(aes())

#Caterpillar plot random slopes Model(3)

pDf.s             <- subset(pDf2, ind=="Slopes")

fixsl_p           <- fixef(fit1)[2]
fixsl_t           <- fixef(fit1)[2] + fixef(fit1)[4]

trt.group         <- as.data.frame(distinct(data[,c(1,2)]))
trt.group$id      <- as.factor(trt.group$id)
trt.group         <- cbind(trt.group, fixsl_p, fixsl_t)

pDf.s             <- full_join(pDf.s, trt.group, by="id")
pDf.s$trt.label   <- ifelse(pDf.s$Treatment==0, "Placebo", "T+S")

for (i in 1:nrow(pDf.s)){
    if(pDf.s$Treatment[i]==0) {pDf.s$y2[i] = pDf.s$y[i] + pDf.s$fixsl_p[i]
    } else {pDf.s$y2[i] = pDf.s$y[i] + pDf.s$fixsl_t[i]
      }
}

p3  <- ggplot(pDf.s, aes(reorder(id, y2),y2)) + coord_flip()
p3  <- p3 + facet_wrap(~ ind)
p3  <- p3 + xlab("Patient ID") + ylab("Individual predictions")
p3  <- p3 + theme(legend.title=element_blank())
p3  <- p3 + geom_hline(yintercept=c(fixsl_p, fixsl_t), linetype=c(1, 2))
p3  <- p3 + geom_errorbar(aes(ymin=y2-ci, ymax=y2+ci,
            linetype=factor(trt.label)), width=0)
p3  <- p3 + geom_point(aes())

#Combine plot p1 and p2 using the multiplot function below
multiplot <- function(..., plotlist=NULL, file, cols=1, layout=NULL) {
  library(grid)

  # Make a list from the ... arguments and plotlist
  plots <- c(list(...), plotlist)

  numPlots = length(plots)

  # If layout is NULL, then use 'cols' to determine layout
```

```
  if (is.null(layout)) {
    # Make the panel
    # ncol: Number of columns of plots
    # nrow: Number of rows needed, calculated from # of cols
    layout <- matrix(seq(1, cols * ceiling(numPlots/cols)),
                     ncol = cols, nrow = ceiling(numPlots/cols))
  }

  if (numPlots==1) {
    print(plots[[1]])

  } else {
    # Set up the page
    grid.newpage()
    pushViewport(viewport(layout = grid.layout(nrow(layout),
ncol(layout))))

    # Make each plot, in the correct location
    for (i in 1:numPlots) {
      # Get the i,j matrix positions of the regions that contain this
subplot
      matchidx <- as.data.frame(which(layout == i, arr.ind = TRUE))

      print(plots[[i]], vp = viewport(layout.pos.row = matchidx$row,
                                      layout.pos.col = matchidx$col))
    }
  }
}

multiplot(p1, p2, cols=2)

#Model for age (grand-mean centered), BMI (grand-mean centered) and
menopausal status

#Recode Menopausal status to 0-1
data$MenoStat2 <- ifelse(data$MenoStat==1, 0, 1)

#Grand-mean center Age and BMI
data$Age_c <- data$Age - mean(data$Age)
data$BMI_c <- data$BMI - mean(data$BMI)

fit2  <- lmer(SexualFunction~ StudyPeriod*Treatment +
                Age_c + BMI_c + MenoStat2 +
                (1 +StudyPeriod|id),
              data=data, REML=FALSE, na.action=na.omit)
summary(fit2)

#Deviance difference test
anova(fit1, fit2)

###############################################
# R-code for analyses performed in Section 3.2#
###############################################

#Linear effect (Include EventCount)
fit3  <- lmer(SexualFunction~ StudyPeriod*Treatment +
                EventCount +
                (1 +StudyPeriod|id),
              data=data, REML=FALSE, na.action=na.omit)
summary(fit3)
```

```r
#Random slope EventCount significant?
fit3a  <- lmer(SexualFunction~ StudyPeriod*Treatment +
                 EventCount +
                 (1 + StudyPeriod + EventCount |id),
               data=data, REML=FALSE, na.action=na.omit)
summary(fit3a)

#Deviance difference test
anova(fit3, fit3a)

#Quadratic effect (EventCount + I(EventCount^2))
fit4  <- lmer(SexualFunction~ StudyPeriod*Treatment +
                EventCount + I(EventCount^2) +
                (1 +StudyPeriod|id),
              data=data, REML=FALSE, na.action=na.omit)
summary(fit4)

#Percentage of periods that exceed the 8 events
counts <- data %>% group_by(id,StudyPeriod) %>% summarise(Freq=n())
counts <- as.data.frame(counts)
maxc   <- subset(counts, counts[,3]>8)
perc   <- nrow(maxc)/nrow(counts)

#Random quadratic effect
fit4a  <- lmer(SexualFunction~ StudyPeriod*Treatment +
                 EventCount + I(EventCount^2) +
                 (1 +StudyPeriod+(EventCount+ I(EventCount^2))|id),
               data=data, REML=FALSE, na.action=na.omit)
summary(fit4a)
#Model does not converge

#Interactions with study period
fit5  <- lmer(SexualFunction~ StudyPeriod*Treatment +
                (EventCount + I(EventCount^2))*StudyPeriod +
                (1 +StudyPeriod|id),
              data=data, REML=FALSE, na.action=na.omit)
summary(fit5)

#Deviance difference test model fit5 with model fit4
anova(fit4, fit5)


###############################################
# R-code for analyses performed in Section 3.3#
###############################################

#Function to calculate person means
#Input:
  #data = data set
  #group_by = the variable that indicates the person or group (id)
  #X = variable of which the means needs to be calculated

person.mean <- function(data, group_by, X){
  id.means  <- ddply(data, group_by, function(x, ind){mean(x[,ind],
                     na.rm=TRUE)}, X)
  id.col    <- which(colnames(data)==group_by)
  n.rows    <- as.vector(table(data[,id.col]))
  y.id.mean <- t(as.vector(0))

  for (i in 1:nrow(id.means)){
        v         <- rep(id.means[i,2], n.rows[i])
```

```r
        y.id.mean <- append(y.id.mean, v)
    }

  y.id.mean   <- as.matrix(y.id.mean[-1],,1)
  return(y.id.mean)
}

#Patient means study period
data$StudyPeriod_id.means <- person.mean(data, "id", "StudyPeriod")

#Center the variable around zero
data$StudyPeriod_id.meansC <- data$StudyPeriod_id.means -
mean(data$StudyPeriod_id.means)

#Average proportion of ATP events
mean(data$StudyPeriod_id.means)

#Run a model with group means of study period x treatment interaction
fit1a  <- lmer(SexualFunction~ StudyPeriod*Treatment + EventCount
               I(EventCount^2) + StudyPeriod_id.meansC*Treatment
               + (1 + StudyPeriod|id), data=data, REML=FALSE,
               na.action=na.omit)
summary(fit1a)

#Patient means of event count
data$EventCount_id.means <- person.mean(data, "id", "EventCount")

#Center the variable around zero
data$EventCount_id.meansC <- data$EventCount_id.means -
mean(data$EventCount_id.means)

#Average of EventCount
mean(data$EventCount_id.means)

#Run a model with group means EventCount

fit4b  <- lmer(SexualFunction~ StudyPeriod*Treatment + EventCount +
               I(EventCount^2) + EventCount_id.meansC
               I(EventCount_id.meansC^2) +
               +(1 + StudyPeriod|id), data=data, REML=FALSE,
               na.action=na.omit)
summary(fit4b)

#Remove objects
rm(list = ls())
```
